# Joint Angle, Range of Motion, Force, and Moment Assessment: Responses of the Lower Limb to Ankle Plantarflexion and Dorsiflexion

**DOI:** 10.1155/2021/1232468

**Published:** 2021-09-18

**Authors:** Ukadike Chris Ugbolue, Chloe Robson, Emma Donald, Kerry L. Speirs, Frédéric Dutheil, Julien S. Baker, Tilak Dias, Yaodong Gu

**Affiliations:** ^1^Faculty of Sports Science, Ningbo University, China; ^2^School of Health and Life Sciences, Institute for Clinical Exercise & Health Science, University of the West of Scotland, South Lanarkshire G72 0LH, UK; ^3^CNRS, LaPSCo, Physiological and Psychosocial Stress, University Hospital of Clermont-Ferrand, CHU Clermont-Ferrand, Preventive and Occupational Medicine, WittyFit, Université Clermont Auvergne, 63000 Clermont-Ferrand, France; ^4^Centre for Health and Exercise Science Research, Department of Sport, Physical Education and Health, Hong Kong Baptist University, Kowloon Tong, Hong Kong; ^5^Advanced Textiles Research Group, School of Art and Design, Bonington Building, Nottingham Trent University, UK

## Abstract

There is limited research on the biomechanical assessment of the lower limb joints in relation to dynamic movements that occur at the hip, knee, and ankle joints when performing dorsiflexion (DF) and plantarflexion (PF) among males and females. This study investigated the differences in joint angles (including range of motion (ROM)) and forces (including moments) between the left and right limbs at the ankle, knee, and hip joints during dynamic DF and PF movements in both males and females. Using a general linear model employing multivariate analysis in relation to the joint angle, ROM, force, and moment datasets, the results revealed significant main effects for gender, sidedness, phases, and foot position with respect to joint angles. Weak correlations were observed between measured biomechanical variables. These results provide insightful information for clinicians and biomechanists that relate to lower limb exercise interventions and modelling efficacy standpoints.

## 1. Introduction

The Augmented Video-based Portable System and the gold standard 3D Vicon Motion Analysis System (Vicon-UK, Minns Business Park, West Way, Oxford, UK) are validated motion analyses systems that are useful for evaluating the motion characteristics of the lower limbs [1]. These systems can work simultaneously and may be integrated with force plates and electromyographic systems. From an anatomical perspective and in line with the Vicon® Plug-In-Gait model (which is predominantly used to biomechanically evaluate joint movement), the lower extremity consists of the foot, ankle, knee, and hip joints. These joints all play important roles in human stability and locomotion [2–4]. There is a definitive need in previous and present research for reliable studies focusing upon the differences in ankle, knee, and hip kinematics and kinetics with direct association to dorsiflexion (DF) and plantarflexion (PF).

The ankle and the lower limb joints assist with the transmission of forces and loads between the leg and foot during weight-bearing activities resulting in effective mobility and flexibility [5]. The ankle functionally acts as a talocrural joint with movement controlled by two joints, namely, the distal end of the tibia and fibula of the lower leg and proximal end of the talus of the foot. DF and PF are movements exhibited by the ankle. The subtalar joint along with articulation of the other talar bones facilitates inversion and eversion motions. From a functionality perspective, the dorsiflexor muscles aid the body in clearing the foot during the swing phase as well as controlling plantarflexion of the foot during a heel strike. Plantarflexor muscles are imperative to performance during an abundance of daily tasks, from stair climbing and walking to rising from a chair [6]. During gait motion, the foot must adapt to uneven surfaces and absorb shocks before biomechanically changing to exert force in the later stages when acting as a lever [7]. This further permits the muscles and ligaments to contribute to overall stability, and therefore, a biomechanical evaluation of this function can aid in treatment from injury and notification of any dysfunctions [5, 8].

The normal range of motion (ROM) of the ankle during DF is 10-20 degrees (°) and 25-30° during PF, contributing to inversion and eversion [9]. Among healthy and physically active individuals, greater passive DF ROM is seen to increase the risk of knee displacement and these biomechanical factors can be associated with further injuries such as anterior cruciate ligament injury during landing [10]. Similarly, Flanagan et al. suggest the capabilities of the ankle in relation to the plantarflexors can change as much as 25% depending on age and gender with respect to torque and ROM. Ankle injuries are the most common injury when participating in exercise and sport [4] with the plantarflexors and dorsiflexors potentially playing a role in reducing the likelihood of injury. The ankle plantarflexors can substantially change the risk of an anterior cruciate ligament and knee displacement injury [10]. Conversely, dorsiflexors cannot withstand the same volume of shock as their counterparts. As the ground reaction force applied to the joint increases, the hip, knee, and ankle acquire further strain, which would occur during landing from height or pivotal situations.

The knee is able to passively affect the DF and PF angles at the ankle through the Achilles tendons becoming contracted or relaxed on potentially compromising surfaces [11]. In doing so, the femoral condyles within the knee are appropriately loaded with forces prior to transfer onto the tibial plateau. The loads placed upon the medial and lateral compartments of the tibial plateau are dependent on the structure of the knee and whether varus and valgus deformities are present [9]. If an infirmity becomes apparent such as osteoarthritis, this particular link of the process can be compromised changing the overall mechanical structure of the ankle-subtaler joint complex [12]. The knee also further assists in proprioception through complex neuromuscular structures allowing stability and motion of the lower limbs.

The hip works as a ball and socket joint with motion occurring in three dimensions. The hip joint has two ligaments which anteriorly stabilise the hip (iliofemoral and pubofemoral) and one ligament which is used for posterior stability (ischiofemoral). This allows the femur to flex between 120 and 135° (90° when fully extended) and extend between 10 and 30° [13]. Females generally have a greater ROM and flexibility compared to men due to bone structure differences, related to childbearing. The hip is able to cope with day to day physical activities such as ascending stairs (40-70° of flexion needed) and sit to stand (104° of flexion needed) [14]. Arnold et al. suggest that the hip flexors do not necessarily directly affect the passive movement of the ankle; however, it does facilitate the knee to complete its movement, which can influence the flexibility of the ankle [15].

To date, no research has effectively measured the kinematics and kinetics of the lower limbs during ankle DF and ankle PF in a standing position. Nor has a longitudinal study been completed to incorporate changes over time under DF and PF conditions. Therefore, further in-depth investigation is required. There has been a definitive need, previously and presently for reliable research studies to focus on the differences in ankle, knee, and hip kinematics and kinetics with direct association to PF and DF. There are research topics on DF and PF during landing and other movements; however, there is little information regarding the dynamic movements of the ankle whilst standing in relation to the biomechanical correlates of movement at the knee and hip joints.

The purpose of this study was to investigate any differences in joint angles, range of motion (ROM), forces, and moments between the left and right limbs at the ankle, knee, and hip joints during dynamic DF and PF movements in both male and female subjects. It is hypothesized that (a) females will have a greater ROM in both DF and PF in comparison to males and (b) the heel raise task will produce higher ground reaction forces, higher forces at the lower limb joints, and larger moments at the lower limb joints in comparison to the fore-foot raise task.

## 2. Method

### 2.1. Participants

Twenty-two healthy individuals, twelve males and ten females, with a mean ± SD age, height, and body mass of 23.04 ± 2.8 years, 169.82 ± 5.88 cm, and 69.83 ± 15.03 kg (males: 23.64 ± 3.26 years, 171.77 ± 8.72 cm, and 72.24 ± 16.89 kg; females: 22.18 ± 2.64 years, 163.45 ± 5.19 cm, and 62.75 ± 15.14 kg), respectively, volunteered to participate in the study. All participants were physically active and able-bodied whilst being free from any lower limb injuries at the time of testing. Additional information from the participants regarding activity levels and time of activities before embarking on the experimental protocol was controlled prior to data collection. All participants were required not to have engaged in any physical activity for 48 hours before commencement of the experiment. All participants were right leg dominant. This was confirmed by identifying what leg the subjects preferred to kick a football with. All subjects were tested at the same relative time (morning testing) to minimize data contamination from diurnal variation effects.

Ethical approval was obtained from the University of the West of Scotland (Ethical Approval Number 2017-0967-844). Each participant reviewed the information sheet, completed a medical health questionnaire, and provided written informed consent. There was no obligation from the individuals to complete the study, and participants were given freedom to withdraw at any time. A risk assessment was also completed prior to experimental testing.

### 2.2. Instrumentation and Laboratory Configuration

Eight Vicon Bonita Motion Analysis cameras (Oxford Metrics Ltd, Oxford, UK) mounted on scaffolding provided a field of view that allowed the lower limb movements to be captured for analysis. The Vicon Bonita Motion Analysis System was linked to an ultra giganet box which collected the lower limb kinematic data at a rate of 250 Hz. The two AMTI force plates (1.2 m) (AMTI, Watertown, MA, USA) were connected to the Vicon Bonita Motion System and interfaced via the ultra giganet box. Kinetic data were collected at a sampling rate of 1000 Hz. Prior to data capture, the reset button was activated as data were collected from both systems integrated through the Vicon Nexus software.

### 2.3. Experimental Design

The experimental protocol for this pilot study required one visit per participant to the Biomechanics Laboratory at the University of the West of Scotland. Prior to testing, reflective objects in the laboratory were removed or concealed to prevent interference during the data capture session. Cameras positioned within the field of view of the participants were inspected, and any reflections identified were masked using the Vicon Nexus Mask Cameras software function. The Vicon Bonita Motion Analysis system was both statically and dynamically calibrated before each session of testing. Prior to testing using the Vicon Nexus software, individual participant details, i.e., age (years), and anthropometric measurements, i.e., height (cm) and body mass (kg) alongside right and left leg length (mm), right and left knee width (mm), and right and left ankle width (mm), were obtained. Body mass and height were measured using a calibrated scale (Seca 803, England) and stadiometer (Seca 213, England). All other measurements were obtained using a small anthropometer measurement instrument-Lafayette (http://ProHealthcareProducts.com, Lehi, UT, USA) and clinical measuring tape. All acquired measurements were imputed into the Vicon Nexus software to define each participant before running the Lower Limb static and dynamic Plug-In-Gait models. The Plug-In-Gait model is the Vicon Nexus' implementation of the conventional gait model. Using a direct (nonoptimal) pose estimation, the Plug-In-Gait computes and defines the position and orientation of each segment based on a set of three tracking markers.

**Figure 1:**
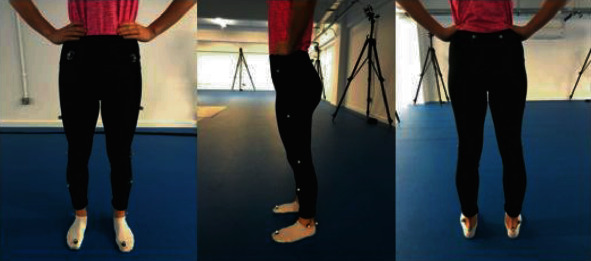
Visual representation of marker placement—positioning of sixteen retroreflective markers on the lower limbs of a participant.

Individuals were required to wear dark, figure-fitting clothing and remove shoes during the session. A technical assistant with experience using the Plug-In-Gait marker instrumentation then placed sixteen (14 mm) retroreflective markers accurately on each individuals' lower limbs according to the Plug-In-Gait marker placement guide. A double-sided tape was used to ensure adherence of retroreflective markers to the skin and fitted clothing of the limb joints and segments. The placements on both the left and right limbs were as follows: anterior superior iliac spine (ASIS), posterior superior iliac spine (PSIS), lateral midthigh, lateral knee, lateral midtibia, lateral malleolus, and calcaneus and 2^nd^ metatarsal head. See Figure 1 for visual representation.

**Figure 2:**
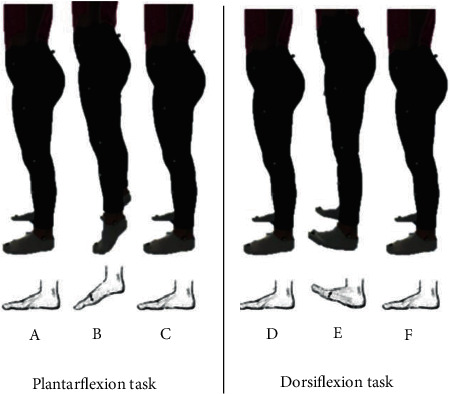
Pictorial description of the plantarflexion (PF) and dorsiflexion (DF) tasks showing (a) loaded phase (foot flat), (b) unloaded phase (during PF), (c) reloading of the heel (foot flat), (d) loaded phase (foot flat), (e) unloaded phase (during DF), and (f) reloaded phase (foot flat). Note both tasks were performed as two independent sessions and were randomised.

Prior to commencement of the dynamic trials, a familiarisation period of ten minutes was permitted to ensure participants were able to complete the movements at a controlled speed. They were only allowed five repetitions for each movement to reduce the potential impact and learning effects of the protocol as outlined by behavioural learning theory. Although the order of the tasks was randomised, to standardize the test protocol, the number of trials, experimenter, and stance were kept consistent throughout each session. Following this, each participant stood on the AMTI force plates, ensuring that the feet were a shoulder width apart and that the hands were placed on the hips. The left and right feet were positioned on two independent force plates. Both feet were placed at the centre and fully within the boundaries of each force plate. Two static trials were taken in this position whilst the participant stood still, followed by two sets of three dynamic trials—DF and PF (Figure 2). The first dynamic trial involved the completion of the DF movement phase. This consisted of three continuous phases being held for two seconds each: foot flat, fore-foot raise, and foot flat. The trial ended after a two-second time limit once the second foot flat phase elapsed. On completion, a further two trials of data were collected. The second set of dynamic trials followed a similar pattern but in a PF movement phase. This consisted of three continuous phases being held for two seconds each: foot flat, heel raise, and foot flat. The trial ended after two seconds of the second foot flat phase. This was repeated twice to ensure that three sets of data were collected. Once tasks were completed, the participant then stepped off the force plates and removed the retroreflective markers and their involvement was complete.

### 2.4. Statistical Analysis

Statistical analysis software Jamovi (Version 0.9.5.12) was used to determine descriptive and inferential statistics. Kinematic (joint angle and ROM) data were manually transferred from the Vicon Nexus 2.8.1 into Microsoft Excel 2019 version 16.23 (Microsoft Corporation, Redmond, Washington, USA). The normality of distribution was determined using the Shapiro-Wilk test. Anthropometric statistical differences between gender and sidedness were examined. Both PF ROM and DF ROM were calculated as follows:

(1) $$\begin{array}{c} {\text{ROM}}_{\left(\text{ankle},\text{knee},\text{hip}\right)}=\left(\text{unloaded phase}–\text{loaded phase}\right)+\left(\text{unloaded phase}–\text{reloaded phase}\right). \end{array}$$

The ROM represented the lower limb range of motion (range of forces and moments was also calculated similarly). The loaded phase represented the joint angle/force/moment at the initial foot flat position, unloaded phase represented the joint angle/force/moment at the fore-foot raise (or heel raise) position, and reloaded phase represented the joint angle/force/moment at the final foot flat position.

General linear model multivariate analyses were applied to the joint angle, joint force, joint moment, and ROM datasets using IBM SPSS Statistics for Windows, Version 25.0. (IBM Corp., Armonk, New York). The within subject variables (dependent variables) were joint angle position, joint force position, joint moment position, and joint angle ROM in the anterior/posterior (*X*), medial/lateral (*Y*), and vertical (*Z*) directions at the ankle, knee, and hip joints. External moments were reported and together with the force outputs were extracted from the Plug-in-Gait model. The between subject factors (independent variable) included gender, sidedness, phases, and foot position. The Pearson correlation (*r*) was performed to establish the level of interaction between the foot position independent variable and the joint angle position, joint force position, joint moment position, and joint angle ROM-dependent variables. When implementing the Pearson correlation, the *r* values obtained varied between −1 and +1 where 1 is a perfect correlation and 0 represents no correlation. Further interpretations of *r* include the following: 1 > *r* ≥ 0.8 (very strong), 0.8 > *r* ≥ 0.6 (moderate), 0.6 > *r* ≥ 0.3 (fair), and 0.3 > *r* ≥ 0.1 (poor) [16, 17]. To determine the effect size, the partial eta squared statistic (*η*_*p*_^2^) in relation to multivariate analyses was calculated. The values of 0.0099, 0.0588, and 0.1379 were considered small, medium, and large effect sizes, respectively [18]. A Bonferroni post hoc test was applied to test for multiple comparisons in the dependent variables for observed means with respect to gender, sidedness, phases, and foot position. *p* < 0.05 was considered significant.

## 3. Results

**Figure 3:**
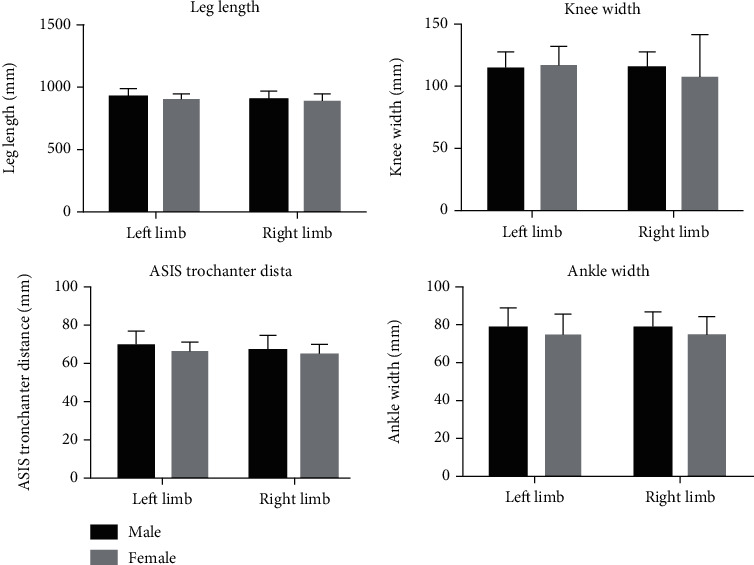
Grouped bar chart representation of the anthropometric datasets with respect to sidedness and gender.

The leg length (mm), anterior superior iliac spine trochanter distance (mm), knee width (mm), and ankle width (mm) anthropometric measurements showed no statistical differences between the left and right limbs (*p* > 0.05) or between genders (*p* > 0.05). The anthropometric measurements in terms of gender and sidedness are displayed in (Figure 3). The ROM at the ankle, knee, and hip joints (Table 1) together with the range of forces (Table 2) over the anteroposterior (*X*), mediolateral (*Y*), and longitudinal (*Z*) axes and resultant datasets are displayed in (Table 3).

In terms of DF movement, the female ankle ROM on the *X*- and *Y*-axes for both limbs were higher in comparison to the males (left female: *X* = 20.610° ± 5.409, *Y* = 0.320° ± 1.606; left male: *X* = 20.067° ± 6.204, *Y* = –0.995° ± 0.571; right female: *X* = 23.030° ± 7.604, *Y* = 1.400° ± 6.511; right male: *X* = 21.989° ± 6.782, *Y* = –0.346° ± 0.332). Females displayed a decreased ROM compared to males in the *X*- and *Z*-axes of the knee joints (left female: *X* = 2.420° ± 2.615, *Z* = 6.890° ± 1.262; left male: *X* = 9.276° ± 2.658, *Z* = 13.957° ± 4.875; right female: *X* = 1.200° ± 2.408, *Z* = 3.810° ± 3.016; right male: *X* = 2.958° ± 3.111, *Z* = 4.378° ± 14.776) and on the *X*-axis of the hip joint for both limbs (left female: *X* = 23.880° ± 4.229; left male: *X* = 30.176° ± 13.597; right female: *X* = 22.850° ± 4.558; right male: *X* = 26.775° ± 7.242).

**Figure 4:**
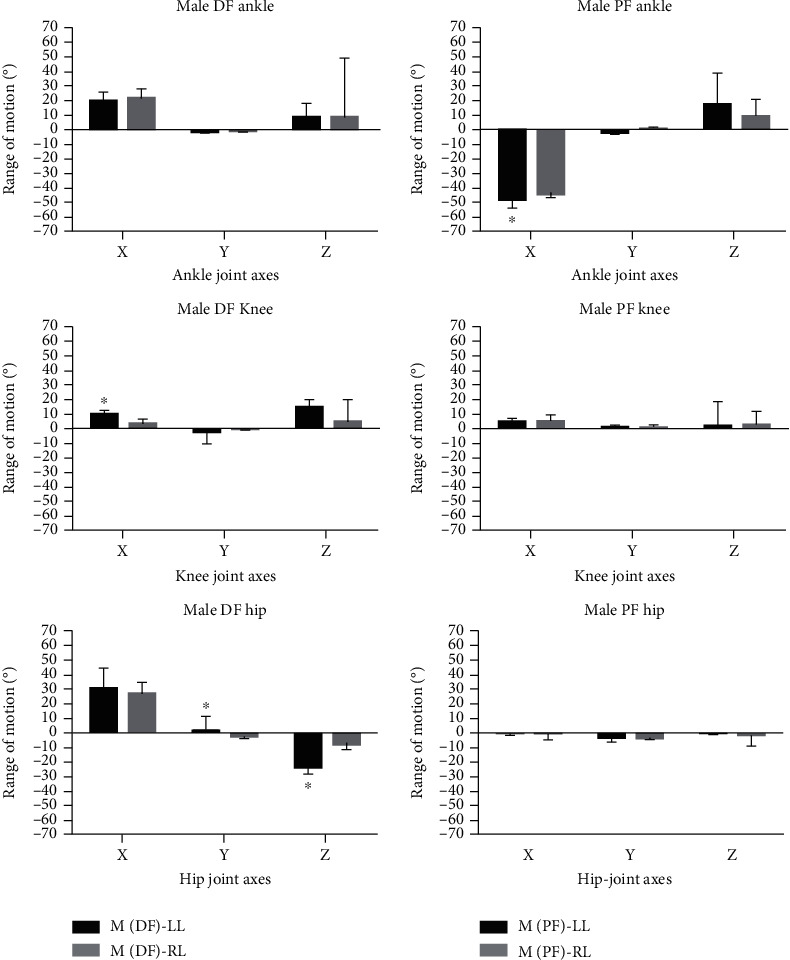
Grouped bar chart representation showing dorsiflexion (DF) and plantarflexion (PF) position comparisons between the left limb (LL) and right limb (LL) in male (M) participants at the anterior/posterior (*X*), medial/lateral (*Y*), and vertical (*Z*) directions at the ankle, knee, and hip joints. Error bars included ±standard deviation whilst ∗ indicates statistical significance (*p* < 0.05) (*n* = 12).

**Figure 5:**
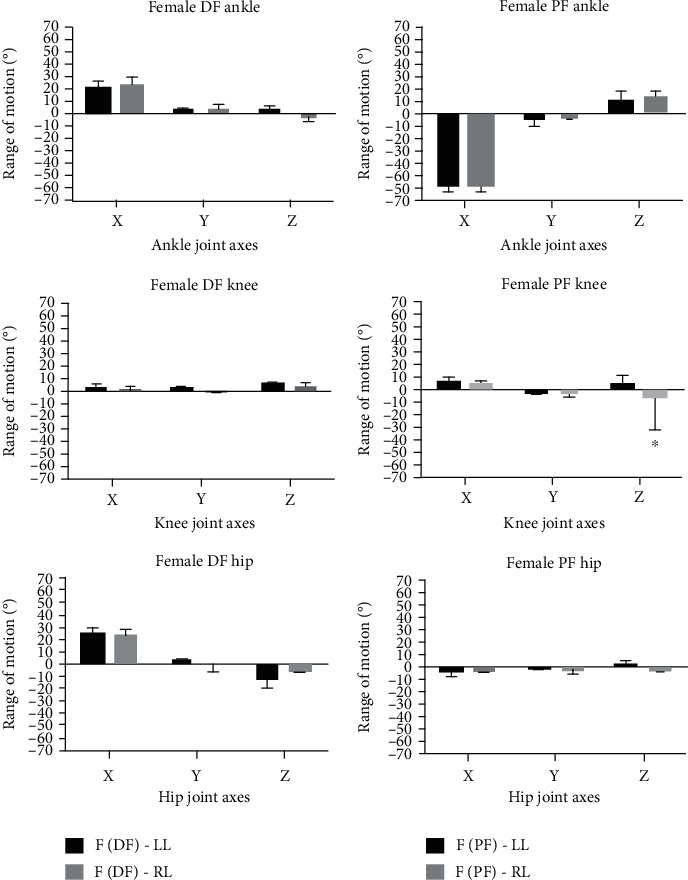
Grouped bar chart representation showing dorsiflexion (DF) and plantarflexion (PF) position comparisons between the left limb (LL) and right limb (LL) in female (F) participants at the anterior/posterior (*X*), medial/lateral (*Y*), and vertical (*Z*) directions at the ankle, knee and hip joints. Error bars included ±standard deviation whilst ∗ indicates statistical significance (*p* < 0.05) (*n* = 10).

Similar to the DF trials, females produced a higher ROM at the ankle over the *X* and *Y* components for both the left and right limbs during PF (left female: *X* = –57.42° ± 3.505, *Y* = –2.79° ± 7.245; left male: *X* = –46.238° ± 5.932, *Y* = –1.853° ± 2.0493; right female: *X* = –57.47° ± 2.888, *Y* = –1.68° ± 0.356; right male: *X* = –42.586° ± 2.652, *Y* = 0.317° ± 0.406). Females displayed an increased ROM compared to males at the *Y*-axis for knee joints (left female: *X* = –0.77° ± 0.561; left male: *X* = 0.822° ± 1.806; right female: *X* = 0.77° ± 1.6304; right male: *X* = 0.696° ± 1.645) and at the *X*-axis of the hip joint for both limbs (left female: *X* = –3.13° ± 5.01; left male: *X* = –0.253° ± 2.436; right female: *X* = –3.2° ± 1.541; right male: *X* = –0.356° ± 7.242). Bar chart displays for males and females in terms of differences in sidedness with respect to DF and PF are shown in Figures 4 and 5, respectively.

The multivariate analysis for the joint angle results showed that there was a significant main effect for gender (*F* = 18.273, *p* < 0.001, *η*_*p*_^2^ = 0.415, large), sidedness (*F* = 2.681, *p* = 0.006, *η*_*p*_^2^ = 0.094, medium), phases (*F* = 5.031, *p* < 0.001, *η*_*p*_^2^ = 0.163, large), and foot position (*F* = 22.112, *p* < 0.001, *η*_*p*_^2^ = 0.462, large). There were also significant main effects for gender and sidedness interactions (*F* = 3.931, *p* < 0.001, *η*_*p*_^2^ = 0.132, medium), as well as phases and foot position interactions (*F* = 11.787, *p* < 0.001, *η*_*p*_^2^ = 0.313, large). No significant differences were observed for interaction effects between gender and phases (*F* = 0.298, *p* = 0.998, *η*_*p*_^2^ = 0.011, medium); gender and foot position (*F* = 1.025, *p* = 0.421, *η*_*p*_^2^ = 0.038, small); sidedness and phases (*F* = 1.000, *p* = 1.000, *η*_*p*_^2^ = 0.007, small); sidedness and foot position (*F* = 0.197, *p* = 0.994, *η*_*p*_^2^ = 0.008, small); gender, sidedness, and phases (*F* = 0.029, *p* = 1.000, *η*_*p*_^2^ = 0.001, small); gender, sidedness, and foot position (*F* = 0.085, *p* = 1.000, *η*_*p*_^2^ = 0.003, small); gender, phases, and foot position (*F* = 0.243, *p* = 1.000, *η*_*p*_^2^ = 0.009, small); sidedness, phases, and foot position (*F* = 0.151, *p* = 1.000, *η*_*p*_^2^ = 0.006, small); and gender, sidedness, phases, and foot position (*F* = 0.047, *p* = 1.000, *η*_*p*_^2^ = 0.002, small).

The between-subjects effect yielded a significant effect for gender with respect to the ankle joint angle in the *Y* direction (*F* = 12.522, *p* < 0.001, *η*_*p*_^2^ = 0.050, small); hip joint angle in the *Y* direction (*F* = 17.339, *p* < 0.001, *η*_*p*_^2^ = 0.067, medium); knee joint angle in the *X* direction (*F* = 17.025, *p* < 0.001, *η*_*p*_^2^ = 0.066, medium); and knee joint angle in the *Y* direction (*F* = 16.250, *p* < 0.001, *η*_*p*_^2^ = 0.063, medium). Sidedness also yielded a significant effect for the ankle joint angle in the *Y* direction (*F* = 5.542, *p* = 0.019, *η_p_*^2^ = 0.023, small) and hip joint angle in the *Z* direction (*F* = 6.884, *p* = 0.009, *η*_*p*_^2^ = 0.028, small). Significant effects were observed for phases regarding the ankle joint angle in the *X* direction (*F* = 17.785, *p* < 0.001, *η*_*p*_^2^ = 0.129, medium) and hip joint angle in the *X* direction (*F* = 10.190, *p* < 0.001, *η*_*p*_^2^ = 0.078, medium). The foot position also produced significant effects with respect to the ankle joint angle in the *X* direction (*F* = 103.015, *p* < 0.001, *η*_*p*_^2^ = 0.300, large); and hip joint angle in the *X* direction (*F* = 23.412, *p* < 0.001, *η*_*p*_^2^ = 0.089, medium). Apart from the gender and sidedness interaction (for the knee in the *X* direction (*F* = 10.482, *p* = 0.001, *η*_*p*_^2^ = 0.042, small) and hip joint angle in the *Y* direction (*F* = 6.649, *p* = 0.011, *η*_*p*_^2^ = 0.027, small)); and phases and foot position interaction (for the ankle joint angle in the *X* direction (*F* = 105.157, *p* < 0.001, *η*_*p*_^2^ = 0.467, large) and hip joint angle in the *X* direction (*F* = 13.271, *p* > 0.001, *η*_*p*_^2^ = 0.100, medium)); no significant effects were observed for all other interactions.

The multivariate analysis for the ROM results only produced a significant effect for foot position (*F* = 82.448, *p* < 0.001, *η*_*p*_^2^ = 0.912, large). All other main effects and interactions were not significant, i.e., sex (*F* = 0.788, *p* = 0.628, *η*_*p*_^2^ = 0.090, medium); sidedness (*F* = 1.793, *p* = 0.084, *η*_*p*_^2^ = 0.183, large); gender and sidedness interaction (*F* = 0.178, *p* = 0.996, *η*_*p*_^2^ = 0.022, small); sex and foot position interaction (*F* = 1.772, *p* = 0.089, *η*_*p*_^2^ = 0.181, large); sidedness and foot position (*F* = 1.168, *p* = 0.328, *η*_*p*_^2^ = 0.127, medium); and gender, sidedness, and foot position (*F* = 1.030, *p* = 0.425, *η*_*p*_^2^ = 0.114, medium).

No significant between-subject effects were observed for gender; gender and sidedness interaction; and gender and foot position interaction, with respect to the dependent variables (*F* < 2.013, *p* > 0.05, *η*_*p*_^2^ = 0.054, small). The following between-subject effects with respect to ROM produced significant effects, namely: sidedness at the knee in the *Z* direction (*F* = 6.981, *p* = 0.010, *η*_*p*_^2^ = 0.080, medium) and at the hip in the *Y* direction (*F* = 5.801, *p* = 0.018, *η*_*p*_^2^ = 0.068, medium); sidedness and foot position at the hip in the *Z* direction (*F* = 5.880, *p* = 0.018, *η*_*p*_^2^ = 0.068, medium); and gender, sidedness and foot position at the knee in the *Z* direction (*F* = 4.545, *p* = 0.036, *η*_*p*_^2^ = 0.054, small). With the exception of knee ROM in the *X* direction (*F* = 0.902, *p* = 0.345, *η*_*p*_^2^ = 0.011, small) and knee ROM in the *Y* direction (*F* = 0.188, *p* = 0.666, *η*_*p*_^2^ = 0.002, small), all other dependent variables showed significant between-subject effects.

The multivariate analysis for the joint force results only produced a significant effect for sidedness (*F* = 9.944, *p* < 0.001, *η*_*p*_^2^ = 0.278, large); phases (*F* = 1.751, *p* = 0.029, *η*_*p*_^2^ = 0.063, medium); foot position (*F* = 5.334, *p* < 0.001, *η*_*p*_^2^ = 0.171, large); and phases and foot position interaction (*F* = 5.097, *p* < 0.001, *η*_*p*_^2^ = 0.164, large). All other main effects and interactions were not significant.

No significant differences between-subject effects were observed for gender; phases; gender and sidedness interaction; gender and phase interaction; gender and foot position interaction; sidedness and phase interaction; gender, sidedness, and phase interaction; gender, sidedness, and foot position interaction; gender, phase, and foot position interaction; sidedness, phase, and foot position interaction; and gender, sidedness, phase, and foot position; with respect to the dependent variables (*F* < 3.486, *p* > 0.05, *η*_*p*_^2^ < 0.014, small). The following between-subject effects with respect to joint force produced significant effects, namely: sidedness at the knee in the *Y* direction (*F* = 70.281, *p* < 0.001, *η*_*p*_^2^ = 0.227, large) and at the hip in the *Y* direction (*F* = 23.066, *p* < 0.001, *η*_*p*_^2^ = 0.088, medium); foot position at ankle in the *Z* direction (*F* = 34.919, *p* < 0.001, *η*_*p*_^2^ = 0.127, medium) and at the knee in the *X* direction (*F* = 6.810, *p* = 0.010, *η*_*p*_^2^ = 0.028, small); sidedness and foot position at the ankle in the *Y* direction (*F* = 13.820, *p* < 0.001, *η*_*p*_^2^ = 0.054, small); and phase and foot position interaction at the ankle in the *Z* direction (*F* = 38.501, *p* < 0.001, *η*_*p*_^2^ = 0.243, small).

The multivariate analysis for the joint moment results only produced a significant effect for gender (*F* = 3.313, *p* = 0.001, *η*_*p*_^2^ = 0.114, medium); sidedness (*F* = 6.237, *p* < 0.001, *η*_*p*_^2^ = 0.195, large); phases (*F* = 3.708, *p* < 0.001, *η*_*p*_^2^ = 0.125, medium); foot position (*F* = 2.395, *p* = 0.013, *η*_*p*_^2^ = 0.085, medium); gender and sidedness interaction (*F* = 4.189, *p* < 0.001, *η*_*p*_^2^ = 0.140, large); and phase and foot position interaction (*F* = 2.482, *p* = 0.001, *η*_*p*_^2^ = 0.087, medium). All other main effects and interactions were not significant.

Only the ankle resultant ROM and force range during PF produced greater ROM and force range in comparison to DF. A converse trend was observed for the ankle moment range datasets (see Table 3). All other resultant joint angle ROM outputs at the knee and hip produced greater DF ROM in comparison to PF. Presented in Table 3 are also the knee and hip resultant force and moment range measurements with respect to gender. The joint angles, forces, and moments produced a similar trend in the Pearson correlation measurements. The correlation between the foot position and the joint angle, joint force, and joint moment-dependent variables ranged from *r* = −0.411 (fair) to *r* = 0.315 (fair). Similarly, the foot position and the ROM dependent variables ranged from *r* = −0.887 (very strong) to *r* = 0.065 (poor).

## 4. Discussion

This study investigated the joint angle, joint force, joint moment, and ROM responses of the left and right ankle, knee, and hip lower limb joints between genders whilst performing dynamic DF and PF movements. Specifically, only the ankle joint during the DF and PF movement produced a significantly (*p* < 0.001) higher PF ROM in comparison to the DF movement. The knee and hip joints showed inconsistencies in their ROM patterns with respect to sidedness and foot position. No observed significant (*p* > 0.05) main effects and interactions for ROM were observed for gender, sidedness, and their combined interactions with foot position. Significant main effects for both joint forces and joint moments were observed for sidedness, phases, foot position, and phases and foot position interaction.

The results suggest that there are no clear definitive distinctions in the ROM coordinate output measures. Our results showed that female participants presented with greater DF and PF ROM when compared to males. This agrees with our first hypothesis. All participants showed different joint angles and ROM output measurements between limbs. This finding may be aligned with joint flexibility since both males and females exhibited variable joint angles and ROM output measurements. Both genders showed a similar trend in the resultant ankle joint ROM during DF and PF for both the left and right limbs. The left and right hip joints also produced similar resultant ROM trends. Although the knee and hip joints produced similar resultant ROM trends for the left limb, the left and right knee produced dissimilar trends. On average, the correlations between the foot position (DF and PF) and the lower limb joint angle and ROM outputs were weak, i.e., poor to fair.

Our joint motion and ROM analysis revealed findings, which differed to expected results. As shown in Tables 1–3, there are no clear distinctions that show that the average ROM was greater in females compared to males; therefore, our results are not in complete agreement with the limited literature available [19, 20]. This could be due to the movement strategies exhibited by participants, which may have subconsciously been influenced by their peculiarities in motion and/or vocational tasks differing in certain ways. ROM required on a daily basis at the ankle joint is reduced below potential; walking requires 30°, and climbing stairs 37° is for ascent and 56° for descent [21]. Similar to Roaas and Andersson, ROM at the *X*-axis of the ankle for both the left and right limb in males and females are higher in PF than in DF; however, they do not significantly differ from each other statistically [22]. The average ROM for all participants in the current study falls within the ROM grouping outlined by the previous study (DF between 5 and 40°; PF between 10 and 55°), suggesting that between the ages 19 and 40 years the plantarflexors and dorsiflexors remain in similar functionality. However, it must also be noted that the toe flexor strength within the foot plays a key role in standing and walking and therefore are independent contributors to future incidence of falls. Performance in a PF trial may be affected by a decrement in toe flexor strength which continues to decline over time [23]. The left and right limb knee joint moment and hip joint forces and moments produced similar trends. In terms of gender, the kinetic outputs from the female participants were not in agreement with our second hypothesis; however, there was a partial agreement in the knee joint forces and moments with respect to the male participants.

Females exhibited higher DF than PF measurements for the left limb knee joint moments, left limb hip joint forces, and left limb hip joint moments. A similar trend was observed for the right limb knee and hip joint forces and joint moments. The males exhibited similar trends; however, differences were observed in the knee joint forces and in the left and right limb joint moments which produced lower DF outputs in comparison to the PF measurements. There are multiple possibilities as to why lower limb DF and PF ROM, forces, and moments may be decreased. These include, but are not limited to, age, gender, prior injury, degenerative diseases, and immobilisation. For example, a traumatic brain injury and fractures are both, in addition to others, known to decrease the compliance of the calf muscles and therefore inhibit full flexibility [24]. Geographic and cultural living conditions are also determinants which can affect ROM [21].

Mechanical stress exerted on the body and the physiological adaptations of tissue over time must also be considered prior to analysing results as this can be apparent with increasing age [10]. Although age was not a focus of this study, it has been suggested to be a confounding factor in the ROM of an individual and a potential reason for primary differences [25]. Wojcik et al. confirm the ideology that younger individuals have a substantially larger ROM compared to older adults as they are more able to utilise the available flexibility [26]. It is also thought that post 50 years of age, muscle strength begins to deteriorate which can progress into reduced motor control at 60+ years with reaction speeds and movements also deteriorating [27]. Individuals, especially the elderly, may also consider strengthening dorsiflexors and plantarflexors to maintain stability, power, and strength necessary for physical function which decreases linearly with age [10, 28]. Similarly, children 5 years and below responded with a decreased hip and knee movement compared to middle aged individuals due to the inability to complete full extension [25]. This is comparable to the decrease in mobility and flexibility in older adults.

Gender-related disparity can arise due to muscle recruitment that is used repeatedly; for example, during a fall, a female is thought to be able to stabilise/recover quicker using ROM and flexibility than males [26]. There are a few limitations in this study that need consideration. Firstly, the participants recruited were aged between 19 and 30, which mean the results are not representative of the entire population. Secondly, due to the limited research in this field, comparisons of results at the ankle, knee, and hip joints were unable to be confirmed or discussed in detail. Therefore, in future studies, a larger sample size needs to be recruited.

Earlier studies by Ugbolue et al. have designed, developed, and validated a new methodological approach to evaluate heel pad stiffness and soft tissue deformation in both limbs between males and females during ankle dynamic unloading and loading activity [29]. To complement Ugbolue et al.'s works [29–32], this study provides a useful biomechanical database from which potential modelling information could be obtained. This will aid in computer simulation designed to provide further understanding and insights into the sensitivity of treatment planning, prediction of treatment outcome, and other healthcare-related opportunities worthy of attention. In general, the force and moment results presented in Table 3 appear small and could suggest that DF and PF exercises may have low impacts in terms of forces exerted on the knee and hip joints. In order to predict treatment outcomes, ankle DF and PF may be a useful rehabilitation exercise regime that could reduce further risks of injuries to the knee and hip among patients recovering from injuries in these specific areas. This research project contributes importantly to the literature and scientific experimentation available on the biomechanics of the lower limbs by exploring the kinematic and kinetic responses to ankle DF and PF. Furthermore, the biomechanical dataset derived from DF and PF movements has been obtained from a healthy population. The data obtained will need to be compared to patient populations to investigate further the clinical usefulness of the kinematic and kinetic parameters presented here. Finally, the results from this study provide insightful information for clinicians and biomechanists that relate to lower limb exercise interventions and modelling efficacy standpoints.

## Figures and Tables

**Table 1:** X, Y and Z ROM joint angle output at the ankle, knee and hip joints for both left and right limbs during the dynamic phases of the trial (°).

Limb sidedness	Joints	Joint co-ordinates	Female DF (mean ± SD) (°)	Female PF (mean ± SD) (°)	Male DF (mean ± SD) (°)	Male PF (mean ± SD) (°)	All participants DF (mean ± SD) (°)	All participants PF (mean ± SD) (°)
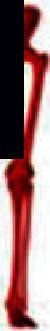 Left limb	Ankle	X	20.610 ±5.409	-57.420 ±3.505	20.067 ±6.204	-46.238 ±5.932	20.310 ± 5.735	-51.320 ± 5.072
		Y	0.320 ±1.606	-2.790 ±7.245	-0.995 ±0.571	-1.853 ±2.0493	-0.400 ± 1.210	-2.280 ± 5.111
		Z	1.360 ± 2.575	10.710 ± 6.653	8.751 ± 9.631	16.647 ± 20.459	5.390 ± 7.293	13.950 ± 15.810
	Knee	X	2.420 ±2.615	6.090 ± 2.854	9.276 ±2.658	4.543 ±1.806	6.160 ± 2.689	5.250 ± 2.343
		Y	0.520 ± 2.008	-0.770 ±0.561	-2.069 ± 8.278	0.822 ±1.806	-0.890 ± 6.309	0.100 ± 0.535
		Z	6.890 ±1.262	3.280 ± 6.957	13.957 ±4.875	1.686 ± 16.203	10.740 ± 3.627	2.410 ± 12.617
	Hip	X	23.880 ±4.229	-3.130 ±5.010	30.176 ±13.597	-0.253 ±2.436	27.310 ± 10.337	-1.560 ± 3.785
		Y	0.230 ± 0.482	-1.610 ± 1.646	1.326 ± 10.300	-3.445 ± 3.065	0.830 ± 7.480	-2.610 ± 2.529
		Z	-11.690 ± 7.276	0.970 ± 2.598	-22.223 ± 4.928	-0.163 ± 1.572	-17.430 ± 5.958	0.350 ± 2.049

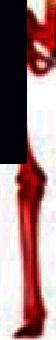 Right limb	Ankle	X	23.030 ±7.604	-57.470 ±2.888	21.989 ±6.782	-42.586 ±2.652	22.460 ± 7.025	-49.350 ± 2.699
		Y	1.400 ±6.511	-1.680 ±0.356	-0.346 ±0.332	0.317 ±0.406	0.450 ± 4.409	-0.590 ± 0.382
		Z	-1.030 ± 3.423	33.120 ± 4.511	8.763 ± 41.224	8.739 ± 11.123	4.310 ± 30.497	19.820 ± 73.176
	Knee	X	1.200 ±2.408	4.940 ± 1.759	2.958 ±3.111	4.839 ± 4.223	2.160 ± 2.796	4.880 ± 3.323
		Y	-0.090 ± 1.098	-0.770 ±1.6304	-0.038 ± 0.887	0.696 ±1.645	-0.060 ± 0.967	0.030 ± 1.617
		Z	3.810 ±3.016	-6.310 ± 26.992	4.378 ±14.776	2.361 ± 8.818	4.120 ± 11.232	-1.580 ± 19.329
	Hip	X	22.850 ±4.558	-3.200 ±1.541	26.775 ±7.242	-0.356 ±7.242	24.990 ± 6.099	-1.650 ± 3.562
		Y	-0.280 ± 4.798	-2.980 ± 2.486	-1.571 ± 2.102	-3.706 ± 1.230	-0.980 ± 3.702	-3.380 ± 1.876
		Z	-5.370 ± 1.165	-2.310 ± 1.120	-6.937 ± 3.490	-1.571 ± 7.817	-6.230 ± 2.638	-1.910 ± 5.799

DF – Dorsiflexion; PF – Plantarflexion; Negative outputs (-) suggest the reloading phase ROM is larger than the loading phase ROM; Positive outputs (+) suggest that the loading phase ROM is larger than the reloading phase ROM.

**Table 2:** X, Y and Z joint range of force output at the ankle, hip and knee joints for both left and right limbs during the dynamic phases of the trial (N).

Limb sidedness	Joints	Joint co-ordinates	Female DF (mean ± SD) (N)	Female PF (mean ± SD) (N)	Male DF (mean ± SD) (N)	Male PF (mean ± SD) (N)	All participants DF (mean ± SD) (N)	All participants PF (mean ± SD) (N)
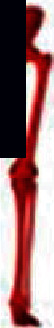 Left limb	Ankle	X	-0.032 ± 0.291	-0.469 ± 0.210	0.270 ± 1.126	-0.591 ± 0.130	0.133 ± 0.859	-0.535 ± 0.172
		Y	0.106 ± 0.154	-0.407 ± 0.086	0.444 ± 0.125	-1.049 ± 0.397	0.290 ± 0.136	-0.758 ± 0.297
		Z	-0.924 ± 0.516	1.653 ± 0.116	-1.017 ± 0.238	1.697 ± 0.236	-0.974 ± 0.393	1.677 ± 0.190
	Hip	X	-0.094 ± 0.298	0.048 ± 0.078	-0.353 ± 0.158	-0.010 ± 0.306	-0.235 ± 0.227	0.016 ± 0.235
		Y	-0.074 ± 0.155	0.058 ± 0.053	0.041 ± 0.088	-0.089 ± 0.124	-0.011 ± 0.120	-0.023 ± 0.100
		Z	-0.040 ± 0.187	-0.286 ± 1.268	-0.657 ± 1.889	-0.071 ± 0.204	-0.377 ± 1.374	-0.169 ± 0.874
	Knee	X	-0.290 ± 0.251	0.214 ± 0.499	-0.265 ± 0.128	0.238 ± 0.483	-0.276 ± 0.191	0.227 ± 0.481
		Y	-0.014 ± 0.259	0.078 ± 0.058	-0.148 ± 0.156	-0.002 ± 0.064	-0.087 ± 0.205	0.034 ± 0.060
		Z	-0.240 ± 0.186	-0.009 ± 0.186	-0.487 ± 1.615	-0.017 ± 0.232	-0.375 ± 1.476	-0.014 ± 0.208

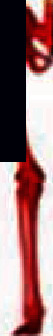 Right limb	Ankle	X	-0.111 ± 0.280	-0.792 ± 0.144	-0.407 ± 1.461	-0.935 ± 0.274	-0.273 ± 1.103	-0.870 ± 0.226
		Y	-0.224 ± 0.369	0.529 ± 0.128	-0.476 ± 0.508	1.500 ± 3.174	-0.361 ± 0.441	1.058 ± 2.358
		Z	-1.028 ± 0.753	1.526 ± 0.173	-1.223 ± 0.162	1.638 ± 0.202	-1.135 ± 0.534	1.587 ± 0.189
	Hip	X	-0.153 ± 0.321	0.038 ± 0.080	-0.388 ± 0.289	-0.103 ± 0.407	-0.281 ± 0.300	-0.039 ± 0.301
		Y	0.076 ± 0.090	0.011 ± 0.142	0.683 ± 2.803	0.165 ± 0.151	0.407 ± 2.076	0.095 ± 0.147
		Z	0.367 ± 1.855	0.062 ± 0.220	0.328 ± 1.215	-1.705 ± 10.494	0.346 ± 1.506	-0.902 ± 7.719
	Knee	X	-0.295 ± 0.271	0.277 ± 0.199	-0.398 ± 0.076	0.269 ± 0.118	-0.351 ± 0.191	0.273 ± 0.156
		Y	0.164 ± 0.296	-0.098 ± 0.085	0.326 ± 0.130	0.059 ± 0.049	0.252 ± 0.216	-0.013 ± 0.67
		Z	0.536 ± 2.096	0.143 ± 0.247	0.351 ± 1.493	0.272 ± 0.227	0.435 ± 1.789	0.213 ± 0.233

DF – Dorsiflexion; PF – Plantarflexion; Negative outputs (-) suggest the reloading phase ROM is larger than the loading phase ROM; Positive outputs (+) suggest that the loading phase ROM is larger than the reloading phase ROM.

**Table 3:** Range of Motion (Joint Angle), Range of Forces and Moments Output – Resultant measurements at the ankle, hip and knee joints for both left and right limbs during the dynamic phases of the trial (°).

Limb sidedness	Joints	Resultant measurement	Female DF (mean ± SD)	Female PF (mean ± SD)	Male DF (mean ± SD)	Male PF (mean ± SD)
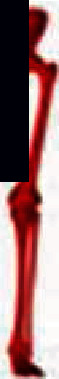 Left limb	Ankle	Joint angle (°)	20.657 ± 6.202	58.477 ± 10.442	21.915 ± 11.470	49.178 ± 21.400
		Joint force (N)	0.931 ± 0.612	1.766 ± 0.255	1.142 ± 1.158	2.081 ± 0.480
		Joint moment (Nmm)	320.248 ± 158.134	203.37 ± 127.766	485.938 ± 881.768	302.093 ± 108.393
	Knee	Joint angle (°)	7.321 ± 3.530	6.960 ± 7.541	16.886 ± 9.968	4.915 ± 16.403
		Joint force (N)	0.126 ± 0.384	0.296 ± 1.272	0.747 ± 1.898	0.114 ± 0.388
		Joint moment (Nmm)	142.932 ± 70.291	57.415 ± 88.639	188.285 ± 127.090	105.423 ± 83.966
	Hip	Joint angle (°)	24.031 ± 8.430	3.871 ± 5.879	37.499 ± 17.755	3.458 ± 3.919
		Joint force (N)	0.290 ±0.406	0.228 ± 0.536	0.574 ± 1.628	0.239 ± 0.540
		Joint moment (Nmm)	45.143 ± 149.963	25.122 ± 53.475	130.911 ± 86.212	138.982 ± 69.468

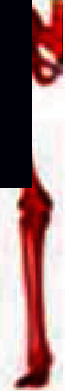 Right limb	Ankle	Joint angle (°)	23.095 ± 10.580	66.352 ± 5.368	23.676 ± 41.779	43.475 ± 11.442
		Joint force (N)	0.336 ± 0.884	1.799 ± 0.259	1.374 ± 1.555	2.410 ± 3.192
		Joint moment (Nmm)	291.668 ± 136.709	184.173 ± 169.349	424.310 ± 158.204	274.117 ± 59.366
	Knee	Joint angle (°)	3.996 ± 4.013	8.051 ± 27.098	5.284 ± 15.126	5.429 ± 9.914
		Joint force (N)	0.405 ± 1.885	0.074 ± 0.274	0.851 ± 3.069	1.716 ± 10.503
		Joint moment (Nmm)	106.293 ± 113.329	54.974 ± 52.011	136.236 ± 144.455	99.029 ± 282.296
	Hip	Joint angle (°)	23.474 ± 6.720	4.945 ± 3.132	27.704 ± 8.309	4.041 ± 10.727
		Joint force (N)	0.633 ± 2.134	0.327 ± 0.328	0.656 ± 1.501	0.387 ± 0.260
		Joint moment (Nmm)	159.135 ± 1076.132	46.801 ± 24.778	107.119 ± 114.153	162.610 ± 113.992

DF – Dorsiflexion; PF – Plantarflexion.

## Data Availability

The biomechanical data used to support the findings of this study are available from the corresponding author upon request.
